# Regular football training down-regulates miR-1303 muscle expression in veterans

**DOI:** 10.1007/s00421-021-04733-1

**Published:** 2021-07-01

**Authors:** A. Mancini, D. Vitucci, F. M. Orlandella, A. Terracciano, R. M. Mariniello, E. Imperlini, E. Grazioli, S. Orrù, P. Krustrup, G. Salvatore, P. Buono

**Affiliations:** 1 Department of Movement Sciences and Wellness, University Parthenope, Naples, Italy; 2 CEINGE-Biotecnologie Avanzate, Naples, Italy; 3 IRCCS SDN, Naples, Italy; 4 University “Foro Italico”, Rome, Italy; 5 Department of Sports Science and Clinical Biomechanics, University of Southern Denmark, Odense, Denmark; 6 Sport and Health Sciences, College of Life and Environmental Sciences, St. Luke’s Campus, University of Exeter, Exeter, UK; 7 Danish Institute for Advanced Study (DIAS), University of Southern Denmark, Odense, Denmark

**Keywords:** Football training, miR-1303, Autophagy, Longevity, BAG-2

## Abstract

**Purpose:**

Regular exercise affects the expression of several genes, proteins and microRNAs (miRNAs) in time- and intensity-dependent manner promoting longevity. We previously identified from GeneChip Array analysis several differentially expressed genes and miRNAs in muscle from veteran football players (VPG) compared to active untrained elderly subjects (CG); here we focussed on miRNA-1303 (miR-1303). The aims of the present research were: to analyse the effects of football training on the expression of miR-1303 and to identify its putative target involved in the longevity pathways in skeletal muscle from VPG compared to CG.

**Methods:**

RNA samples from 12 VPG and 12 CG muscle biopsies were used to validate miR-1303 expression. Crossing four different bioinformatic algorithms, we identified 16 putative targets of miR-1303; from these, BAG-2, KLHL7 and KBTBD6 were chosen for further validation by Western blot analysis in LHCN-M2 human myoblasts transiently transfected with miR-1303.

**Results:**

Football training down-regulates miR-1303 expression in muscle from VPG compared to CG and the expression of BAG-2, a chaperon protein involved in the autophagy pathway, inversely correlated to overexpression of miR-1303 in a time-dependent manner, indicating that it is a miR-1303 potential target.

**Conclusions:**

This is the first report, to our knowledge, describing miR-1303 regulation in skeletal muscle by football training and the identification of a target protein, BAG-2, involved in the autophagy pathway. This result contributes to the enlargement of knowledge on the molecular mechanisms linking football training, autophagy and longevity.

## Introduction

Successful ageing is a multi-domain concept which comprises different biopsychosocial factors that can counteract the progressive decline of biological functions. Although the biological ageing process is unstoppable, it has now been established that regular exercise can counter some of the adverse physiological and cognitive consequences of ageing. In recent years, it has been reported that different genes, proteins and microRNAs (miRNAs) are involved in gene expression regulation during skeletal muscle ageing (Schmidt et al. 2019; Fochi et al. 2020).

MiRNAs are a family of single-stranded, non-coding, short RNA molecules which play a role in cellular metabolism and processes and are emerging as key regulators in gene expression at the post-transcriptional level. We previously performed a GeneChip array analysis to identify differentially expressed genes and miRNAs in skeletal muscles from veteran football players (VPG) compared to active untrained elderly (CG) subjects and we demonstrate that the expression of key messengers involved in the proteasome promotion and autophagy processes are up-regulated in VPG muscle (Mancini et al. 2019). Here, we further analysed Gene chip data, focussing our attention on the miR-1303 MiR-1303 is involved in the tumorigenesis and progression of several cancers as prostate cancer (Bo et al. 2019), neuroblastoma (Li et al. 2016), gastric (Zhang et al.^2014^) and colorectal cancer (El-Murr et al. 2012). Moreover, miR-1303 results up-regulated in patients with type 2 diabetes mellitus (T2DM), and is indicated as a novel biomarker for this pathology (Wang et al. 2016). To date, there are no evidence on miR-1303 and on the effects mediated by exercise training on the expression in skeletal muscle.

Furthermore, some miRNAs, together with heat-shock proteins (HSPs), play an important role in autophagy-associated pathways (Talebian et al. 2020). HSPs are molecular chaperones and have been implicated in longevity and ageing in many species. The autophagy pathway, being a highly regulated process for recycling intracellular protein and organelles, results in up-regulation in response to danger and differentiation signals (Au et al. 2016; Levine 2005; Shintani and Klionsky 2004). Exercise induces the activation of HSPs in skeletal muscle cells (Koh and Escobedo 2004; Paulsen et al. 2009). It has also been demonstrated that not only acute exercise but also long-term recreational training can lead to an increase in HSPs in veteran skeletal muscle (Mancini et al. 2019), together with systemic improvement: anti-oxidative potential, metabolic adaptations and cardiovascular capacity (Alfieri et al. 2015; Andersen et al. 2016; Bangsbo et al. 2015; Krustrup et al. 2010a, b; Krustrup and Krustrup 2018; Mancini et al. 2017; Schmidt et al. 2015).

Within this frame, the aims of the present study were: (a) to analyse the effects of football training on the expression of miR-1303 (b) to identify its putative targets and the possible involvement in the longevity pathways in skeletal muscle from veteran football players (VPG) compared to active untrained elderly subjects (CG).

## Materials and methods

### Enrolment

The samples used for this study are from participants recruited in the ClinicalTrials.gov identifier: NCT01530035. Twenty-four healthy males aged 66–72 years voluntarily participated in the study. 12 VPG and 12 CG matched for age were enrolled. VPG subjects were recruited from local football clubs in the Copenhagen area. The anthropometric and clinical characteristics of the subject participating in the study are reported in Table 1. In detail, VPG had an average age, height and weight, BMI and fat percentage of 69 ± 3 years, 177 ± 3 cm, 78 ± 7 kg, 24.8 ± 2.1 and 22.3 ± 1.5%, respectively, and a maximal oxygen uptake of 35 ± 5 ml/min/kg. On average, they had been active football players for 49 ± 10 years (range: 25–58 years), and for the last 10 years they had attended one football training session per week (1.5 ± 0.6 h/session) and played 26 ± 12 football matches per year (5v5 or 11v11). Two of the 12 footballers in VPG were former elite football players, whereas the remaining 10 were life-long recreational football players. The control group participants were healthy habitually active individuals recruited via ads in local newspapers, with an average age, height, weight, BMI and fat percentage of 68 ± 2 years, 176 ± 4 cm, 83 ± 10 kg, 26.9 ± 3.4, 28.8 ± 1.4%, respectively, and a maximal oxygen uptake of 28 ± 4 ml/min/kg. CG were on average active 2 h/week on average (range 0–9 h/week), mainly with everyday activities such as walking, housing and gardening. The participants in CG had not been involved in regular structured exercise training for a major part of their adult life (Schmidt et al. 2015). Exclusion factors were a history or symptoms of cardiovascular disease or cancer, type 2 diabetes, hypertension, nephropathy or musculoskeletal complaints. The study was conducted in line with the Declaration of Helsinki and was approved by the local research ethics committee in Copenhagen, H-1–2011-013. The subjects’ habitual fitness level was assessed by a questionnaire. All participants signed an informed consent form.

**Table 1:** Anthropometric and clinical characteristics of subjects participating to the study

	VPG	CG
Number of subjects	12	12
Age (yrs)	69 ± 3	68 ± 2
Height (cm)	177 ± 3	176 ± 4
Body weight (kg)	78 ± 7	83 ± 10*
BMI (kg/m^2^)	24.8 ± 2.1	26.9 ± 3.4*
Total body fat (%)	22.3 ± 1.5	28.8 ± 1.4*
VO_2_peak (ml/kg/min)	35 ± 5	28 ± 4*

Value are reported as means ± SD

*p < 0.05 VPG vs CG

### Muscle biopsies

Muscle biopsies were obtained under standardised conditions between 7 and 10 a.m. after an overnight fast, and taken from the vastus lateralis under local anaesthetic (1% Lidocaine, Amgros 742122, Copenhagen, Denmark) using the Bergstrom technique as detailed in Andersen et al. (2016). Briefly, the muscle sample (40 mg wet weight) was immediately frozen in liquid nitrogen and stored at −80 °C pending further analysis. All participants abstained from intense physical activity or training for 48–72 h before the biopsies.

### MiRNA expression profiles in muscle biopsies

Sample RNA extraction was performed as previously described (Mancini et al. 2017). Briefly, total RNA was extracted from the muscle biopsies using a miRNeasy Mini kit (Qiagen, Hilden, Germany) according to the manufacturer’s instructions. The RNA integrity number (RIN) of samples was assessed using a Bio-Rad Experion automated electrophoresis station (Hercules, CA, United States) before cDNA synthesis. 12 RNA samples of VPG and 7 RNA samples CG passed the criterion of RIN > 7. 6 RNA samples from VPG and 6 RNA from CG were used to obtain three pooled VPG (named VPG1, VPG2, and VPG3) and three pooled CGs (named CG1, CG2, and CG3) libraries that underwent to microarray assay to determine gene expression profile, as previously described (Mancini et al 2019). We carried out the profiling using GeneChip R Human Transcriptome Array 2.0 (HTA 2.0, Affymetrix, Santa Clara, CA, United States). The RNA samples were prepared using the WT PLUS Reagent kit, followed by hybridization on HTA 2.0 microarray chips. 100 ng of total RNA were subjected to two cycles of cDNA synthesis with the Affymetrix WT PLUS expression Kit. DNA fragments are then terminally labelled by terminal deoxynucleotidyl transferase (Affymetrix) with biotin. The biotinylated DNA was hybridised to the Human Genechip. HTA 2.0 Arrays (Affymetrix), containing more than 285.000 full-length transcripts covering 44.700 coding genes and 22.800 non-coding genes selected from H. sapiens genome databases RefSeq, ENSEMBL, and GenBank. The data obtained from the GeneChip were deposited in the Gene Expression Omnibus of the NCBI (Edgar et al. 2002) and are accessible through GEO Series Accession Number GSE125830-(Mancini et al. 2019). The validation of miR-1303 was performed on individual samples (12 VPG and 7 CG) by RT*q*PCR (see below).

### Bioinformatic analysis

Computational predictions of the putative targets of miR-1303 were performed using public target-prediction tools with different algorithms: TargetScan 7.0, http://www.targetscan.org; miRDB, http://mirdb.org; microRNA, http://www.microrna.org; MicroT4, http://diana.imis.athena-innovation.gr/DianaTools. As for TargetScan 7.0, we filtered the 4445 putative targets of miR-1303 by setting the TargetScan CS (context score) ≤ -0.4. Similarly, amongst 448 putative targets obtained from miRDB, a minimum cutoff value of 56 was selected for the target SCORE. No filter was applied to screen the list of putative targets obtained by microRNA and MicroT4 tools.

### Cell culture and transient transfection in LHCN-M2

The human skeletal muscle LHCN-M2 cell line, kindly provided by Dr Vincent Mouly (Institut de Myologie, Paris, France) (Zhu et al. 2007), was maintained at a subconfluent density (70%) at 37 °C in 5% CO_2_ in growth medium (GM) as described in Vitucci et al. (2018). Cells were seeded between 60 and 80% confluence in 6-well Petri dishes and cell transfection was performed using according to the manufacturer’s instructions (Thermo Fisher Scientific, Inc., Italy). 5 μl of Lipofectamine™ RNAiMAX Transfection Reagent (Thermo Fisher Scientific, Inc., Italy) was diluted in 250 μl of Opti-MEM medium and 5 μl mirVana^®^ miRNA mimic miR-1303 (ID#: MC13799;GGCUGGGCAACAUAGCGAGACCUCAACUCUACAAUUUUUUUUUUUUUAAAUUUUAGAGACGGGGUCUUGCUCUGUUGCCAGGCUUU) or mirVana™ miRNA Mimic, Negative Control #1 (ID#: 4464058, named miR-CTRL) synthesised by Thermo Fisher Scientific in 250 μl of Opti-MEM^®^ Medium. Subsequently the miR-1303 or miR-CRTL was diluted with Lipofectamine^®^ RNAiMAX Reagent (1:1 ratio) and incubated for 5 min at RT. SiRNA-lipid complex was added to cells which were then incubated for 24 and 48 h at 37 °C in 5% CO_2_.

### RNA extraction and quantitative real-time-PCR (RT*q*PCR)

Total RNA was extracted from the 19 muscle biopsies (*N* = 12 VPG and *N* = 7 CG) and from LHCN-M2 cell lines 24 and 48 h after transfection (n.3 independent experiments for each time point were performed) and integrity assessed as described above. 10 ng of total RNA obtained from individual VPG (n.12) and CG (n.7) muscle biopsies and from the LHCN-M2, were reverted in cDNA using a TaqMan microRNA reverse transcription kit (Thermo Fisher Scientific, Inc., Italy) with specific reverse primer and with the following thermal cycles: 16 °C and 42 °C for 30 min each, followed by 85 °C for 5 min according to the manufacturer’s instructions. Subsequently, the mature form of miRNAs was detected using the miR-1303 primers (ID 002792) and TaqMan Universal Master Mix II purchased from Thermo Fisher Scientific. RNU44 (ID 001094) expression level was used as internal control for the normalisation of miR-1303. For each cDNA, the RT*q*PCR reaction was performed in triplicate with the following thermal cycling parameters: 95 °C for 10 min, followed by 40 cycles of 95 °C for 15 s and 60 °C for 1 min. RNU44 expression level was used as internal control for the normalisation of miR-1303 and the fold changes were calculated using the formula 2^−ΔΔCt^ (Livak et al. 2001).

### Western blotting

Twenty-four muscle biopsies (12 VPG and 12 CG) were mechanically pulverised and protein extraction was performed as previously described (Mancini et al. 2017). Briefly, protein samples (50 μg each) were separated on 4–20% precast gradient polyacrylamide gels (Bio-Rad), transferred to the Hybond ECL nitrocellulose membrane (GE Healthcare, Italy) and checked by Ponceau S staining to verify equal loading. The membranes were immunoblotted using mouse monoclonal antibodies against BAG cochaperone 2 (BAG-2), glyceraldehyde- 3-phosphate dehydrogenase (GAPDH) (1:1000; Santa-Cruz Biotechnology Inc, USA.) and rabbit polyclonal antibodies against kelch repeat and BTB domain containing 6 (KBTBD6) and kelch-like family member 7 (KLHL7) (Abcam, 1:1000). Blots were incubated with appropriate horseradish peroxidase-conjugated secondary antibody and target proteins were visualised by ECL detection (GE Healthcare, Italy). Densitometric measurements were carried out using Quantity One software (Bio-Rad, Italy) as reported elsewhere (Imperlini et al. 2015). GAPDH protein was used to estimate the total amount of loaded proteins. Results were normalised as a percentage of the mean of controls in each membrane.

### Statistical analysis

Group comparisons were examined by means of ANOVA statistical model. Relative miRNA expression was reported as relative quantization (RQ) values, calculated as 2^−ΔΔCt^, where ΔCt is calculated as Ct target gene—Ct housekeeping genes (RNU44 mRNA expression). One-way ANOVA was used to analyse miR-1303 expression in muscle from CG and VPG; relative protein abundance of BAG-2, KBTBD6 and KLHL7, was calculated respect to GAPDH abundance. Differences between VPG *vs* CG were considered statistically significant at *p* < 0.05. Two-way mixed ANOVA [within-subjects factor: time (24 h and 48 h); between-subjects factor: miR-treat (miR-1303 and miR-CTRL)] was performed; in the presence of positive miR-treat × time interaction, Bonferroni post hoc test was carried out. Statistical analysis was performed with StatView software (version 5.0.1.0; SAS Institute Inc., Cary, NC, United States).

## Results

### Identification of differently expressed miRNA in skeletal muscle from VPG and CG

**Fig. 1:**
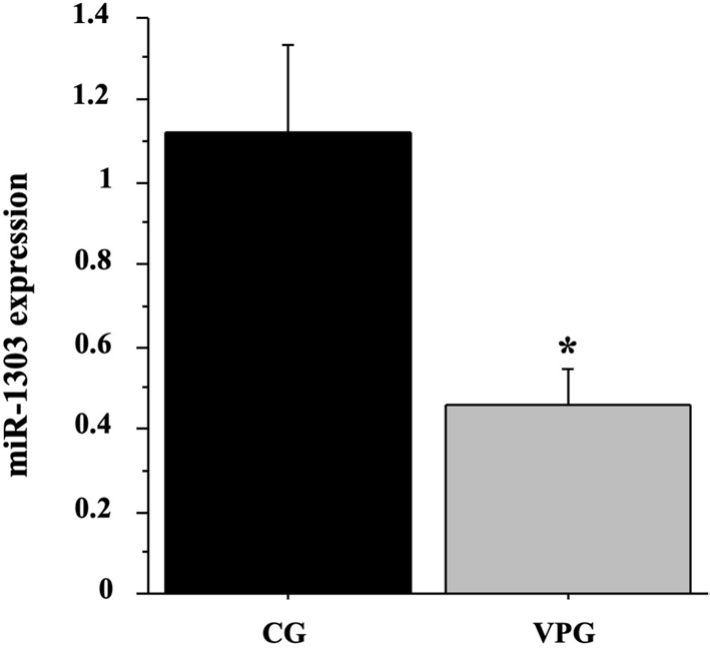
miR-1303 expression in muscle from VPG compared to CG using RT*q*PCR analysis. Quantitative analysis expression of miR-1303 was determined in skeletal muscle biopsies from 7 CG (black bar) and 12 VPG subjects (grey bar). Data represent the mean (± SD) of miR-1303 relative expression in VPG and CG muscle biopsies (2^−ΔΔCT^) and compared by one-way ANOVA; differences were considered significant at *p < 0.05 vs CG

We previously identified the differently expressed genes (DEGs) and miRNAs in skeletal muscle from VPG compared to CG subjects by means of a GeneChip analysis and deposited them in NCBI’s Gene Expression Omnibus (GEO Series Accession Number GSE125830 -Mancini et al. 2019). In particular, we previously demonstrated that messengers associated with autolysosome and the proteasome-mediated pathways were significantly up-regulated in skeletal muscle by VPG compared to CG. Here we focussed on differentially expressed miRNAs in skeletal muscle by VPG versus CG subjects and on their putative interactors. The GeneChip analysis showed that 12 miRNAs were differentially expressed in muscle VPG respect to CG; amongst them, we focussed our attention on miR-1303 (*p* < 0.01). We confirmed the different expression of miRNA- 1303 in the 12 individual VPG compared to 7 CG muscle samples by RTqPCR (*p* < 0.05, Fig. 1).

### Identification of putative miR-1303 targets through bioinformatic algorithms

**Fig. 2:**
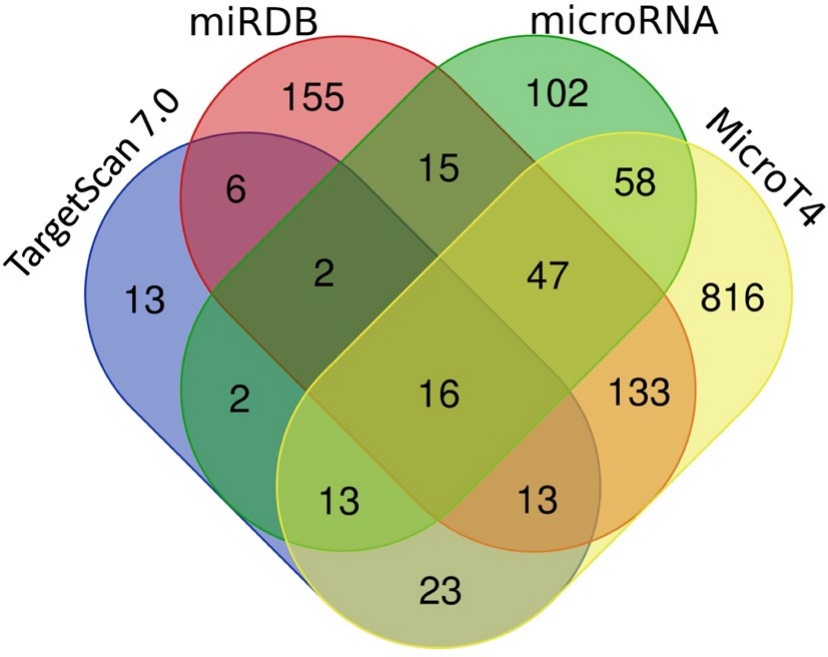
Venn diagram of miR-1303 putative targets. Venn diagram shows overlapping miR-1303 targets specific for each bioinformatic tool: TargetScan 7.0, miRDB, microRNA and MicroT4

The targets of miR-1303 were analysed using public target-prediction tools with different algorithms. In particular, we focussed on the top putative targets identified by TargetScan7.0 (88), miRDB (386), microRNA (255) and MicroT4 (1119), respectively, using cutoff and significance threshold as indicated in “Materials and methods”. Successively, we overlapped the outputs of the four different tools to optimise the in silico target prediction (Fig. 2), thus identifying 16 putative targets of miR-1303 (Table 2).

**Table 2:** miR-1303 putative targets predicted by crossing of four different algorithms: (TargetScan, miRDB, microRNA and MicroT4)

Gene symbol	Protein name
PDIA3	Protein disulfide isomerase family A, member 3
MLF1	Myeloid leukaemia factor 1
KBTBD6	Kelch repeat and BTB (POZ) domain containing 6
KLHL7	Kelch-like family member 7
YWHAZ	Tyrosine 3-monooxygenase/tryptophan 5-monooxygenase activation protein, zeta polypeptide
TNFAIP8L3	Tumour necrosis factor, alpha-induced protein 8-like 3
TMEM40	Transmembrane protein 40
STATH	Zinc finger and BTB domain containing 6
P2RY14	Purinergic receptor P2Y, G-protein coupled, 14
EIF1B	Eukaryotic translation initiation factor 1B
TG	Thyroglobulin
BAG-2	BCL2-associated athanogene 2
VAMP3	Vesicle-associated membrane protein 3
SLC35E3	Solute carrier family 35, member E3
AGBL2	ATP/GTP binding protein-like 2
PAPOLB	Poly(A) polymerase beta (testis specific)

**Fig. 3:**
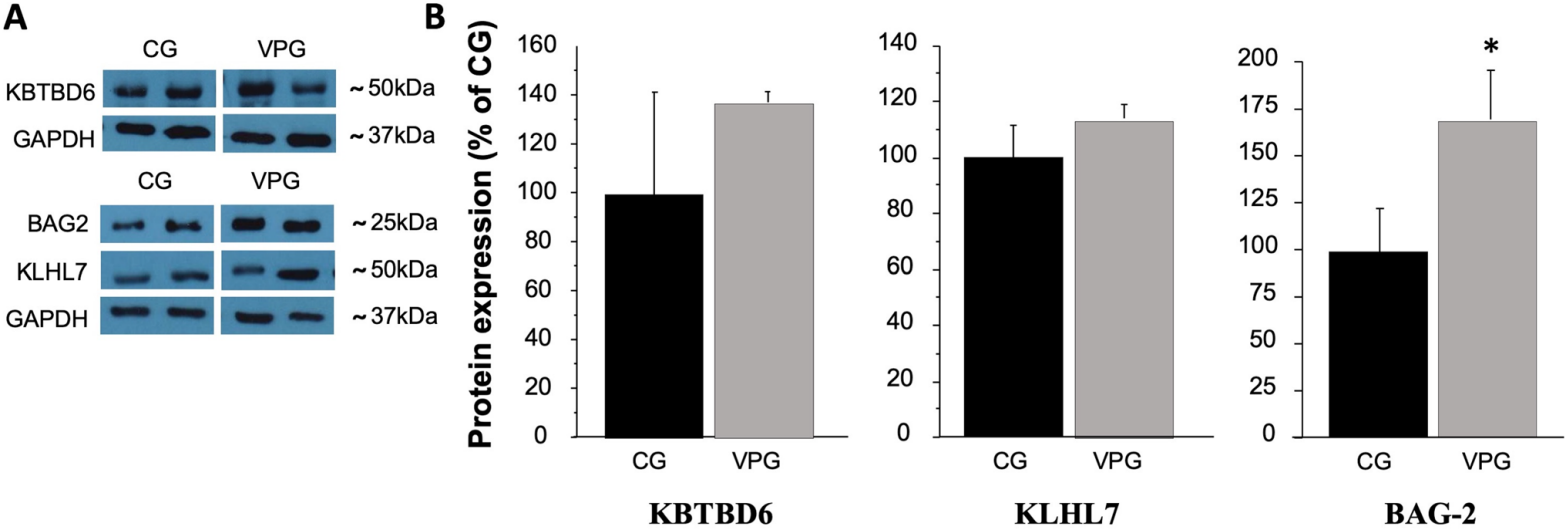
KBTBD6, KLHL7 and BAG-2 protein expression levels in skeletal muscle from CG and VPG. Protein expression levels of KBTBD6, KLHL7 and BAG-2 predicted targets of miR-1303 evaluated by Western Blotting in muscle biopsies from 12 CG (black bars) and 12 VPG (grey bars) and analysed by one-way ANOVA. **A** Representative blots are reported for each protein; **B** data represent the optical densitometry means (± SD) of three different experiments reported as a percentage of CG expression; differences were considered significant at *p < 0.05 vs CG

Amongst predicted target genes, BAG-2, KLHL7 and KBTBD6, involved in the protein quality control proteasome pathway, were chosen for further validation. Interestingly, BAG-2, a chaperon protein involved in preventing disregulated ubiquitination of misfolded protein by CHIP (carboxyl terminus of Hsp70-interacting protein), resulted up-regulated (*p* < 0.05) in skeletal muscle from VPG compared to CG (Fig. 3A, B).

### BAG-2 is the target of miR-1303 in LHCN-M2

**Fig. 4:**
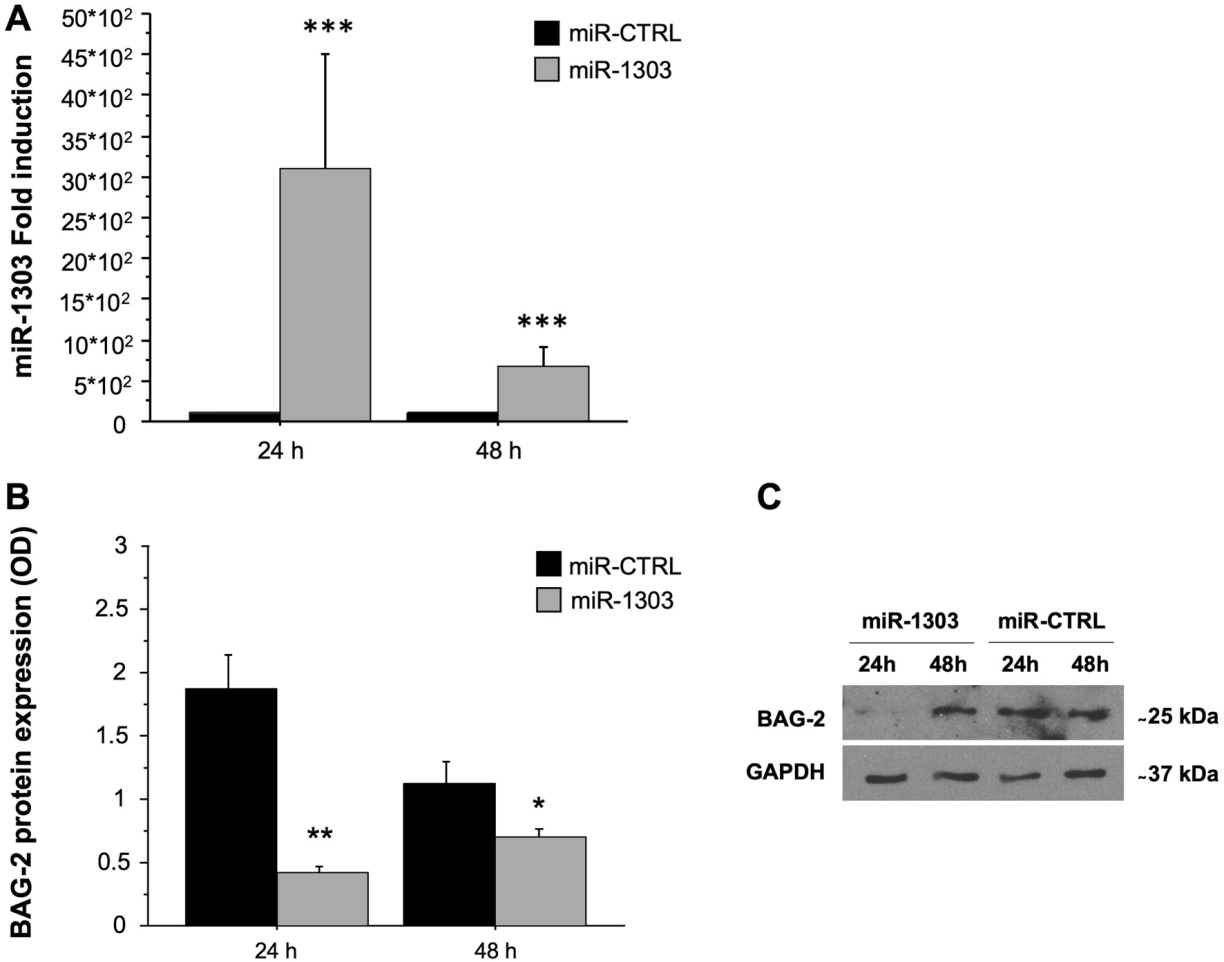
BAG-2 is the target of miR-1303 in LHCN-M2. **A** Quantitative analysis expression of miR-1303 was determined in LHCN-M2 cells. Fold-induction represents miR-1303 compared to miR-CTRL expression after 24 and 48 h of transient transfection. An arbitrary value of 1 was assigned to the expression of miR-CTRL. one-way ANOVA showed significant differences ***p < 0.001 for miR-1303 (grey bars) compared to miR-CTRL (black bars) at 24 and 48 h. **B** Protein expression levels of BAG-2, the target of miR-1303, were analysed by Western Blotting in LHCN-M2 transfected with miR-1303. Data represent the optical densitometry means (± SD) of three different experiments. Differences were considered significant at **p < 0.01 for miR-1303 (grey bars) compared to miR-CTRL (black bars) transfected cells at 24 h and at 48 h *p < 0.05. **C** Representative blots are reported for each protein analysed

To investigate the effect of miR-1303 on BAG-2 protein expression, we overexpressed miR-1303 in human myoblast cell line LHCN-M2; as a negative control miR-CTRL was used. The transfection efficiency was verified by RT*q*PCR after 24 and 48 h (miR-treat effect: *p* < 0.001, Fig. 4A). Two-way mixed ANOVA [within-subjects factor: time (24 h and 48 h); between-subjects factor: miR-treat (miR-1303 and miR-CTRL)] was performed (miR-treat × time interaction: *p* < 0.01) Bonferroni post hoc test was carried out (Fig. 4B, C). One-way ANOVA showed a significant increase of miR-1303 expression at 24 h (*p* < 0.01); the expression was reduced at 48 h albeit higher than miR-CTRL (*p* < 0.05).

## Discussion

The main objectives of this study were to assess the effects of football training on skeletal muscle miR-1303 expression level and to find its putative interactors. We demonstrate that miR-1303 expression was down-regulated in muscle from VPG compared to active untrained elderly subjects and we identify BAG-2, a chaperon protein involved in preventing unregulated ubiquitination of misfolded protein by CHIP, as a down-regulated molecule by miR-1303 in human myoblast LHCN-M2 cells. To the best of authors’ knowledge, this is the first report describing miR-1303 regulation by football training and the identification of the putative target protein.

MiRNAs regulate different cellular processes including proliferation, motility and apoptosis (Bartel 2004). MiRNAs with preferred expression in skeletal muscle are termed “MyomiRs” and regulate muscle development, plasticity and functionality (Güller and Russell 2010; Moresi et al. 2015). Regular exercise promotes positive adaptations in skeletal muscle. In particular, it improves muscle mass, resistance to fatigue and cardiovascular fitness, increasing general quality of life (Petriz et al. 2017; Naseeb et al. 2017; Gries et al. 2018). Several studies have investigated the effects of different types of exercise on miRNA expression (Da Silva et al. 2020; Falzone et al. 2020; Fochi et al. 2020). Only a few studies have investigated the correlation between exercise and miRNA expression in the elderly, particularly in acute or short-term training. A recent meta-analysis evidenced only nine circulating miRNAs differently expressed in young compared to elderly subjects after acute exercise (Margolis et al. 2017; Gopinath et al. 2018).

Regular exercise training mediates the reduction in the risk of chronic non-communicable diseases (NMCT), including cancer (Cartee et al. 2016; Gebel et al. 2015; Kyu et al. 2016) and promotes wellbeing and longevity (Garatachea et al. 2015), also through the regulation of miRNAs and protein expression. In the last few years, adapted sport training, particularly football, has been reported as holistic positive paradigm, linking training to improved cardiovascular, metabolic and musculoskeletal fitness, also in the elderly (Krustrup et al 2010a, 2018; Bangsbo et al. 2015; Andersen et al. 2016; Krustrup and Krustrup 2018; Imperlini et al. 2020). Regular training promotes successful ageing, activating the autophagy process in muscle tissue (Fan et al. 2016). Despite growing evidence linking regular exercise to longevity, the underlying molecular mechanisms are not completely understood. In particular, the effects mediated by football training on miRNA muscle expression associated with longevity, to our knowledge, have not been reported until now. In this context, we provide evidence that miR-1303 expression was down-regulated in skeletal muscle from VPG compared to active untrained elderly subjects. MiR-1303 also plays an important role in cancer development by acting as an oncogene in different types of tumours, such as in neuroblastoma where it promotes proliferation (Li et al. 2016) and in gastric cancer to modulate proliferation and invasion (Zhang et al. 2014), in colorectal cancer (El-Murr et al. 2012) and in prostate cancer progression and development (Liu et al. 2019). In addition, miR-1303 overexpression was associated with microvascular complications in T2DM patients (Wang et al. 2016).

We recently demonstrated that in veteran muscle, the autophagy pathways were enhanced: we found up-expression of Beclin (Bcl-2), ATG (ATG5-ATG12 complex), heat-shock (HSC70/90) and PSMD13 (proteasome complex) proteins, suggesting a more efficient protein quality control process in veteran trained muscle compared to untrained active elderly subjects, which correlates with longevity (Quan and Lee 2013; Walczak et al. 2013; Wedel et al. 2018; Mancini et al. 2019). The maintenance of proteostasis is fundamental for the function and viability of cells. On the other hand, the deterioration of these pathways is associated with several diseases such as Alzheimer’s, Parkinson’s and T2DM; moreover, proteostasis pathway impairment is a hallmark of ageing (Harlt et al. 2011; He and Klionsky 2009; Ross and Poirier 2004). Molecular chaperon proteins, like HSPs, are involved in correct protein-folding and in the autophagic lysosomal pathway (Douglas and Dillin 2010; Lopez-Otin et al. 2013). BAG-2 belongs to a family of 6 BCL2-associated athanogene members (BAGs) (Behl 2016) which are conserved in different non-human, mammal and plant species, suggesting a key biological role in cell physiology (Doukhanina et al. 2006; Takayama and Reed 2001). BAG-2 improves correct protein folding by interacting with HSP70/CHIP complex and prevents aggregations of misfolded proteins (Arndt et al. 2005; Dai et al. 2005; Schönbühler et al. 2016; Wang et al. 2008); furthermore, BAG proteins can bind to various transcriptional factors, to regulate various processes such as cell apoptosis and differentiation (Qin et al. 2016).

Our results indicate that BAG-2 protein is up-regulated in muscle from veterans compared to active untrained elderly subjects, and it is in turn associated with a healthier profile although, up to now, no direct evidence correlating miR-1303 expression and longevity pathways is known. It is worth noting that BAG-2 up-expression in muscle from VPG subjects is in line with the increase of Bcl-2, ATG5-ATG12 complex, HSC70/90 and PSMD13 protein expression involved in the lysosomal and proteasome pathways as we previously reported (Mancini et al. 2019). Therefore, we can speculate that football training positively is related to a healthier profile and longevity promotion in veterans.

In conclusion, we provide evidence, for the first time, that miR-1303 expression and BAG-2 protein, its putative target, are regulated by football training in veterans muscle tissue. We also contribute to enhance the knowledge of molecular mechanisms linking l football training to longevity. The limited number of subjects investigated represents the main limitation of this study, as well as the absence of females. Further elucidation of the effects mediated by different types of sports training on muscle miR-1303 and BAG-2 protein expression will contribute to greater understanding of this process in females too. In the same way, it will be useful to investigate these processes on subjects who have been playing football and/or other types of sports for a shorter time and to discriminate whether these effects are due to the life-long football training or only to football training.

Finally, research into circulating miR-1303 represents a future goal to define healthy ageing biomarkers.
